# A Review of the Australian MRI Linac Program: From Pie in the Sky to Research Milestone

**DOI:** 10.1111/1754-9485.70133

**Published:** 2026-06-13

**Authors:** Michael Bernard Barton, Geoffrey Paul Delaney, Peter Greer, Lois Holloway, Peter Metcalfe, Robba Rai, Trang Pham, William S. Price, David Waddington, Stuart Crozier, Paul Keall

**Affiliations:** ^1^ South Western Campus, School of Clinical Medicine University of New South Wales Sydney New South Wales Australia; ^2^ Ingham Institute for Applied Medical Research Liverpool New South Wales Australia; ^3^ Liverpool and Macarthur Cancer Therapy Centres Liverpool New South Wales Australia; ^4^ Department of Radiation Oncology Calvary Mater Newcastle Newcastle New South Wales Australia; ^5^ School of Mathematical and Physical Sciences University of Newcastle Newcastle New South Wales Australia; ^6^ Centre for Medical Radiation Physics University of Wollongong Wollongong New South Wales Australia; ^7^ Nanoscale Organisation and Dynamics Group, School of Science Western Sydney University Penrith New South Wales Australia; ^8^ Image X Institute, Sydney School of Health Sciences, Faculty of Medicine and Health the University of Sydney Sydney New South Wales Australia; ^9^ The School of Information Technology and Electrical Engineering University of Queensland Brisbane Queensland Australia

**Keywords:** image‐guided radiotherapy, MRI linac, MRI spectroscopy

## Abstract

The Australian Magnetic Resonance Imaging (MRI) Linear Accelerator program (MRI linac) was a major research project that aimed to build and test a unique MRI linac prototype for cancer treatment. It aimed to improve radiotherapy anatomical targeting and explore physiological targeting. The purpose of this report is to summarise the development and achievements of the program so as to provide an example of a successful large‐scale research project in Australian radiation oncology. The project involved six Australian universities and international collaborators. We developed and built a unique MRI linac configuration comprising a 1 T magnetic field and a 6 MV accelerator, with the beam delivered in line with B_0_ and the patient placed across B_0_ in the split between the two halves of the magnet. The broad research domains were personalised disease targeting, medical device innovation, and biodiscovery. Specific projects included Artificial Intelligence image enhancement, radiation dosimetry in high magnetic fields, MRI characterisation of cancer heterogeneity in human tumours, and animal and human studies. Over $27 million was obtained to support the program from competitive sources. The program published over 120 papers and supported 25 PhD completions. The learnings from the Australian MRI linac program are that Australia has world‐class radiotherapy research in physics and engineering, that major projects need a lot of time and a lot of collaboration, and that large, novel radiotherapy projects can attract significant funding and produce significant results.

## 1 Introduction

The Australian Magnetic Resonance Imaging (MRI) Linear Accelerator program (MRI linac) was a major research project that aimed to build and test a unique MRI linac prototype for cancer treatment. It received over $27 million in competitive grant funding, established extensive collaborations, and pioneered the introduction of MRI‐guided radiotherapy in Australia over the period 2009 to 2025.

The purpose of this report is to summarise the development and achievements of the program so as to provide an example of a successful large‐scale research project in Australian radiation oncology.

The original proposal was for a bunker using a retired linac for research purposes. This expanded after a significant boost in funding from the Federal Government Health and Hospital Fund in 2008. The research program drew in collaborators from six Australian universities and several international institutions. The introduction of MRI within the radiation oncology department resulted in the development of skills and experience in the application of MRI to radiation treatment delivery.

### 1.1 Pie in the Sky

In 2000, Liverpool Cancer Therapy Centre was five years old and struggling to attract staff because of its geographic position on the outskirts of Sydney, and was disparaged for its status as ‘new kids on the block’ [1]. There were widespread shortages of radiation therapists and medical physicists.

One strategy to attract and retain staff was to develop an academic program for medical physics built around a research bunker that would provide a resource for medical physics research in a clinical setting. It was originally conceived as a separate bunker containing one linac that was no longer in clinical service. Research could be conducted during normal working hours, and experiments would not have to be dismantled to allow the continuation of clinical treatment the next day. It would also be a useful training resource. It was hoped that this facility would attract academic physicists, engineers, radiation therapists and students and develop its own funding with time. We aimed to develop potential world leadership in one area. A now‐lost email referred to the proposal as ‘Pie in the Sky’. The Liverpool Staff Specialists' Trust Fund allocated $1.5 million to the proposal.

### 1.2 Initial Funding

In 2009, the Health and Hospital Funding (HHF) was created by the Federal Government as a financial stimulus for health and research infrastructure after the Global Financial Crisis. It funded the expansion or creation of many health research institutes. The Ingham Institute for Medical Research at Liverpool received $47 million from the HHF for a five‐story research building, including $7.5 million for a Research Bunker. Nine million was available, therefore, for the project when the radiation oncology trust fund's contribution was included. This changed the potential scope and impact of the project, and we resolved on a research program based on MRI‐guided radiotherapy.

All major advances in radiotherapy in the last two decades have come from improvements in imaging of tumours and normal tissues. These advances include image‐guided radiation therapy [2], intensity‐modulated radiation therapy (IMRT) [3, 4, 5], 4D CT combined with IMRT [6], stereotactic body radiotherapy [7], and improved dose calculation [8]. Clinically, they have resulted in higher tumour doses, better cancer control, and fewer side effects. MRI is the best imaging tool available for radiotherapy guidance. MRI linacs allow exquisite imaging of complex tumours and normal tissue, far exceeding the image quality of X‐ray guidance.

MRI linacs challenge the current paradigm that the tumour is treated as a uniform whole rather than as a dynamic, heterogeneous and evolving complex of tumour cells in an ever‐changing microenvironment. The attraction of MRI‐guided radiotherapy is its ability to perform real‐time anatomical and potentially physiological imaging. MRI offers a broad range of physiological imaging capabilities; e.g., R2* imaging is correlated with hypoxia [9]; diffusion weighted imaging with cellularity [10]; blood‐oxygen‐level dependent (BOLD) contrast imaging with neural activity [11]; and dynamic contrast‐enhanced (DCE) imaging with proliferation, vascular permeability and relative extravascular volume [12]. The physiologic imaging capabilities of the MRI enable a virtual whole tumour biopsy prior to and during each treatment, enabling real‐time adaptive physiological targeting as a unique way to treat cancer. The daily physiological images acquired also offer the opportunity to monitor treatment efficacy and enable rapid changes of treatment plans to improve therapy. The increased accuracy can change how radiation is delivered, enabling higher dose, shorter course treatments that benefit patients and the health system.

The program had three broad research themes:

1. *Personalised Disease Targeting* to exploit and maximise the potential of the MRI linac to improve tumour control and minimise toxicity, develop a translational pathway and investigate the use of the MRI linac to treat diseases other than cancer.
2. *Medical Device Innovation*, to optimise the experimental performance of the MRI linac from an imaging and therapy perspective, develop next‐generation system designs, develop MR‐compatible dosimetry systems, and explore MRI‐guided hadron therapy.
3. *Biodiscovery*, to investigate physiologic targeting capabilities to identify and deliver higher radiation doses to the most treatment‐resistant tumour sub‐volumes, undertake biomarker discovery, and develop nanoscience synergies.

### 1.3 Facility Design

At the time the grant was received, there was one commercial MRI‐guided radiation system available that used a low field magnet and, initially, a cobalt source and two prototype MRI linac systems that used 0.5 Tesla (T) and 1.5 T magnets and 6 MV accelerators [13]. All systems used a beam directed perpendicular to the line of the magnetic field (B_0_). The program team had meetings with these groups, but it was clear that the research opportunities would be limited to participation in later clinical studies as early adopters rather than involvement with hardware development. This would not meet the goal of a physics and engineering‐oriented research program.

**FIGURE 1:**
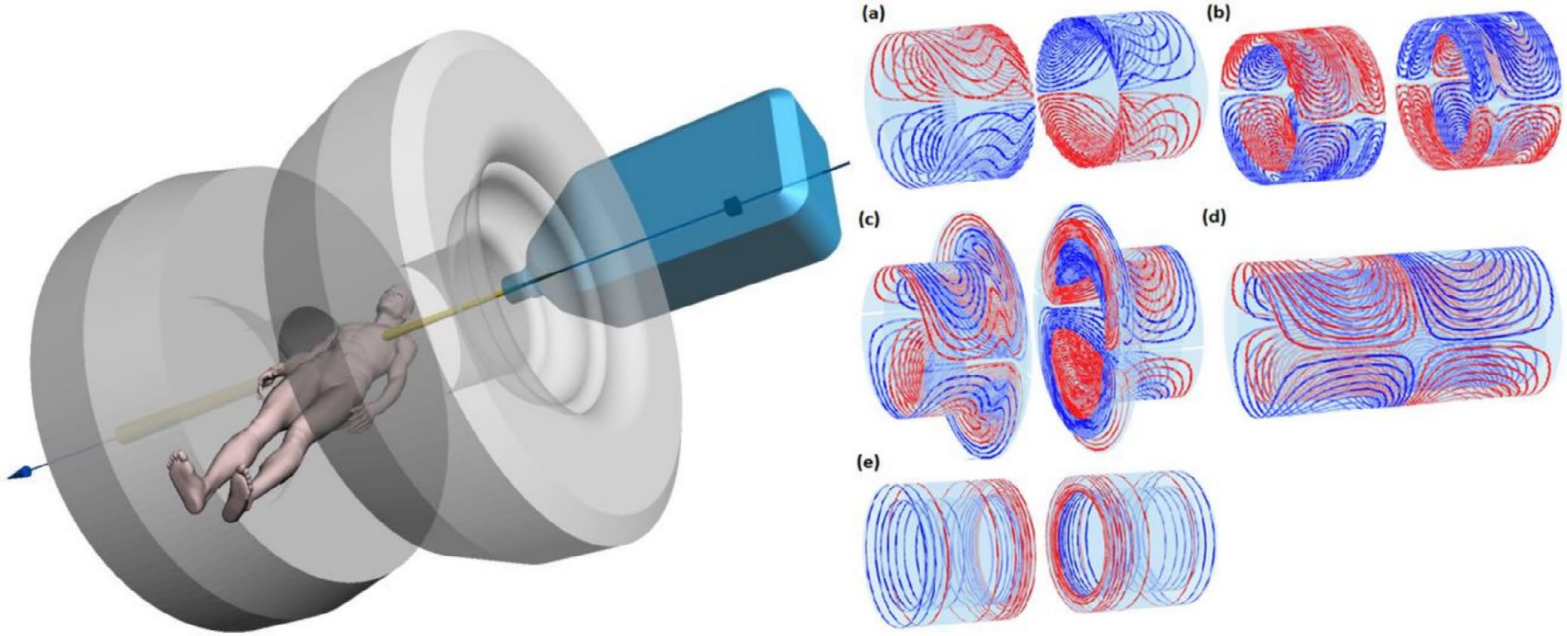
Australian MRI linac concept design (left) [14] (with permission) and field designs for the Australian MRI linac to incorporate the large split between the two halves of the device (right).

We therefore embarked on the development of a unique configuration comprising a 1 T magnetic field and a 6 MV accelerator with the beam delivered in line with B_0_ and the patient placed across B_0_ in the split between the two halves of the magnet [14](Figure 1). The rationale was that beam creation was less affected by B_o_, there were dosimetric advantages, and the design was applicable to particle therapy. Upright image‐guided treatment would also be possible. We recognised the challenge of administering multiple beams, which could only be achieved by patient rotation [15] and there was the likelihood of an increased surface dose from scattered electrons funnelled into the beam [16].

The MRI engineering team at the University of Queensland was internationally recognised for their many contributions to diagnostic MRI design and were approached to collaborate. The MRI hardware aspirations of the system were a split‐bore system capable of being used in‐line or perpendicular orientations to the radiation beam, as high a field strength as possible, as large a gap as possible between the magnets to allow for patient access, and the ability to do fast imaging (pseudo real‐time imaging to track tumours and direct the radiation beam). This essentially meant strong gradient strengths with high slew rates (rapid switching) and the need for parallel imaging to ensure fast imaging. This, in turn, meant both an efficient transmit RF coil and multi‐element receive radiofrequency arrays were needed.

After a number of design studies and engagement with manufacturers (and with a view to the budget), the following high‐level specifications for the imaging system were selected:

- A split‐bore 1 Tesla magnet, 82‐cm diameter bore, 50‐cm gap, actively shielded with a low‐field region along B_0_ where the linac/MLC will be placed (manufactured by Agilent) (Figure 1).
- Spectrometer or control system, MRI subsystem based on Magnetom Avanto (Siemens).
- Gradient coils: High‐performance split‐gradient coil, 50‐cm gap between the coils (manufactured by Tesla).
- RF coils Circularly polarised transmitter coil with an 8‐channel receive array (manufactured by Magnetica).

The radiation beam generation and collimation system for the MRI linac consisted of a Varian Linatron portable 6MV linear accelerator and a Varian Millennium 120 leaf multileaf collimator purchased to provide 6MV photons. The portable linac was chosen to allow variable source to surface distance experiments via a movable table. The portable linac had the potential to allow experiments to be conducted both parallel and perpendicular to B_o_, although the perpendicular configuration was not realised during the program.

### 1.4 Build

**FIGURE 2:**
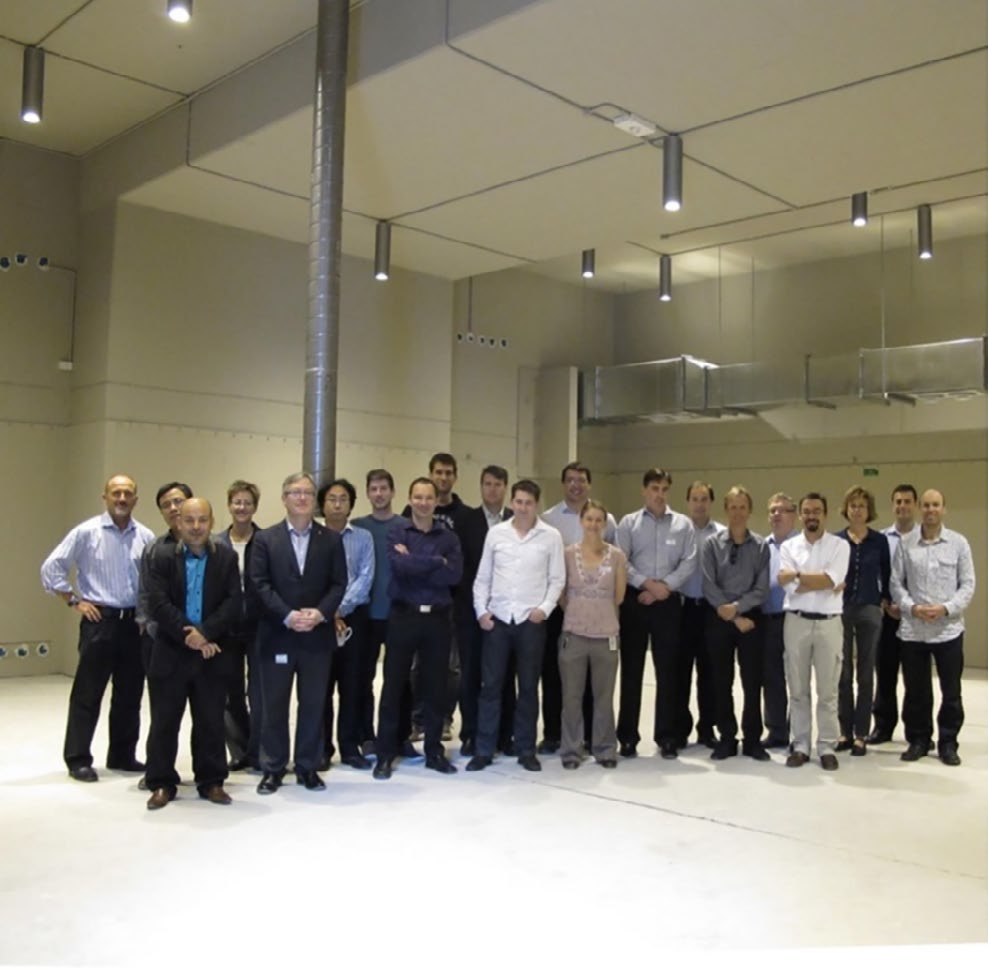
Inside the bunker at Liverpool Hospital before the Faraday cage was installed. The bunker is 90 m^2^, and comprises 600 mm concrete shielding and two primary beam‐stoppers of 1,200 mm concrete. Collaboration team for scale.

The Australian MRI linac required a purpose‐built bunker so that the project would not interfere with clinical services at Liverpool Cancer Therapy Centre. The bunker had to provide shielding for 6MV photons and be large enough to contain a Faraday cage for the MRI and to allow sufficient space for prototype deployment and modification (Figure 2).

**FIGURE 3:**
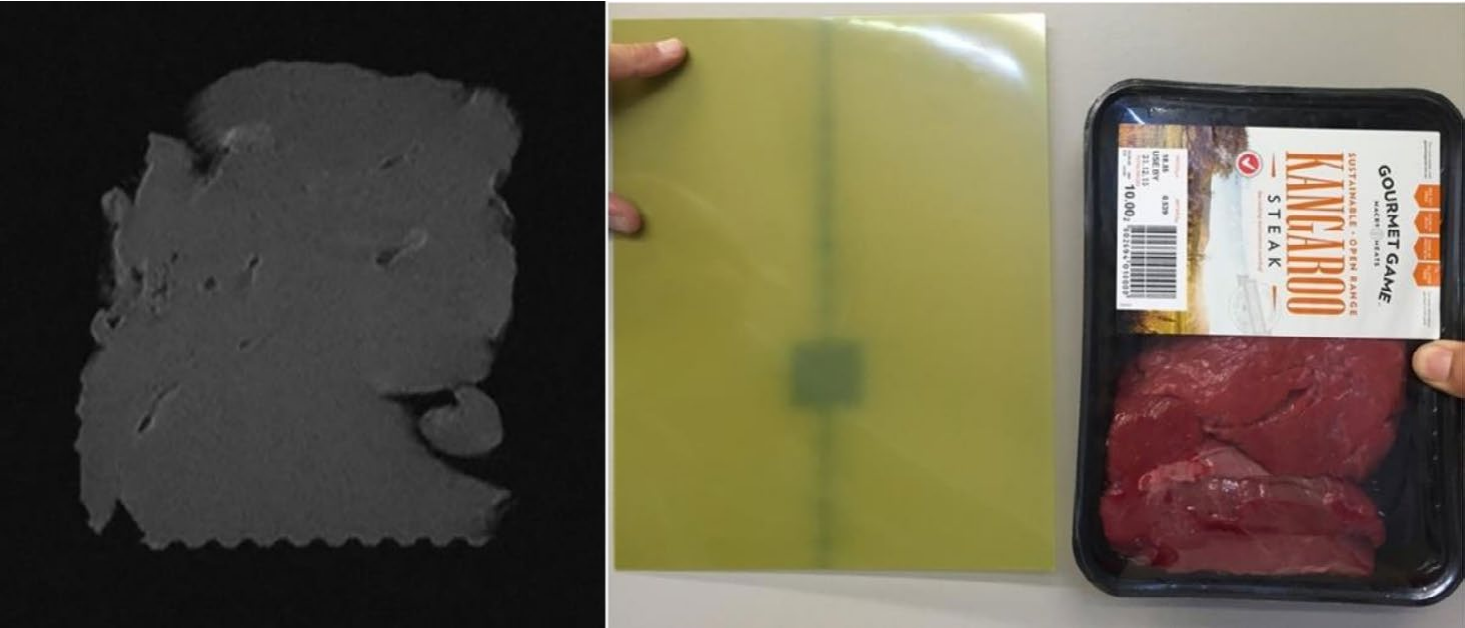
Kangaroo steak (Right) used for the first inline MRI treatment imaging (Left) [18] (with permission).

A decommissioned Siemens 1 T diagnostic MRI (Sonata, Siemens Healthineers) was installed for preliminary experiments while the Agilent split‐bore magnet was being constructed. The Siemens MRI and Linatron provided the first inline experiments in December 2015 [17]. We used a kangaroo steak as the experimental subject (Figure 3), as Utrecht University had used a pork chop for their first‐ever experimental image on the prototype cross‐bore MRI linac [19].

**FIGURE 4:**
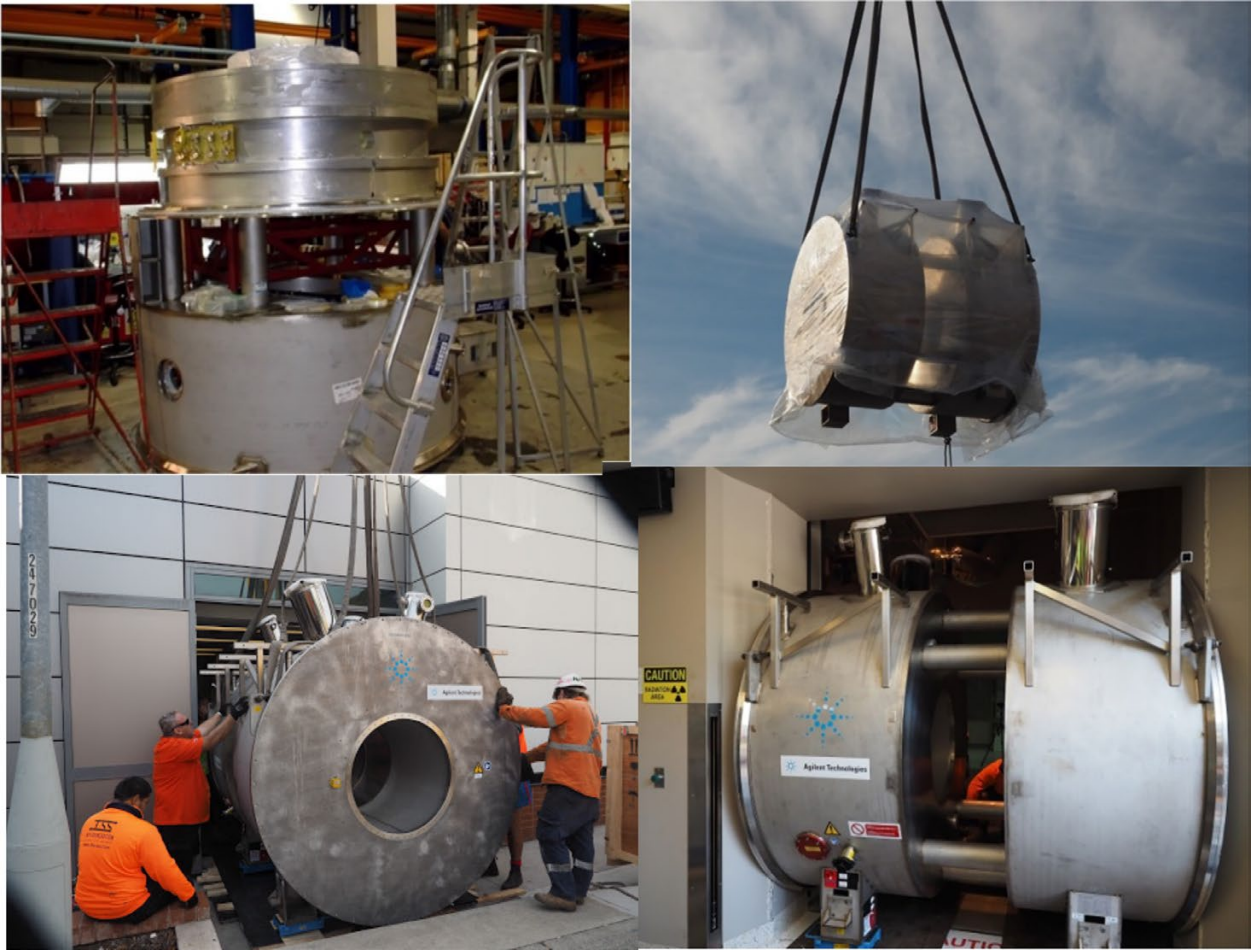
Delivery of Agilent magnet showing completion in the factory, craning, slow movement through to doorways into the bunker. Magnet construction at Agilent (top left), craning the magnet from the delivery truck (top right), manual handling into the building (bottom left), and manual handling through the bunker door (bottom right). There was only 5 cm clearance for entry through the bunker door.

**FIGURE 5:**
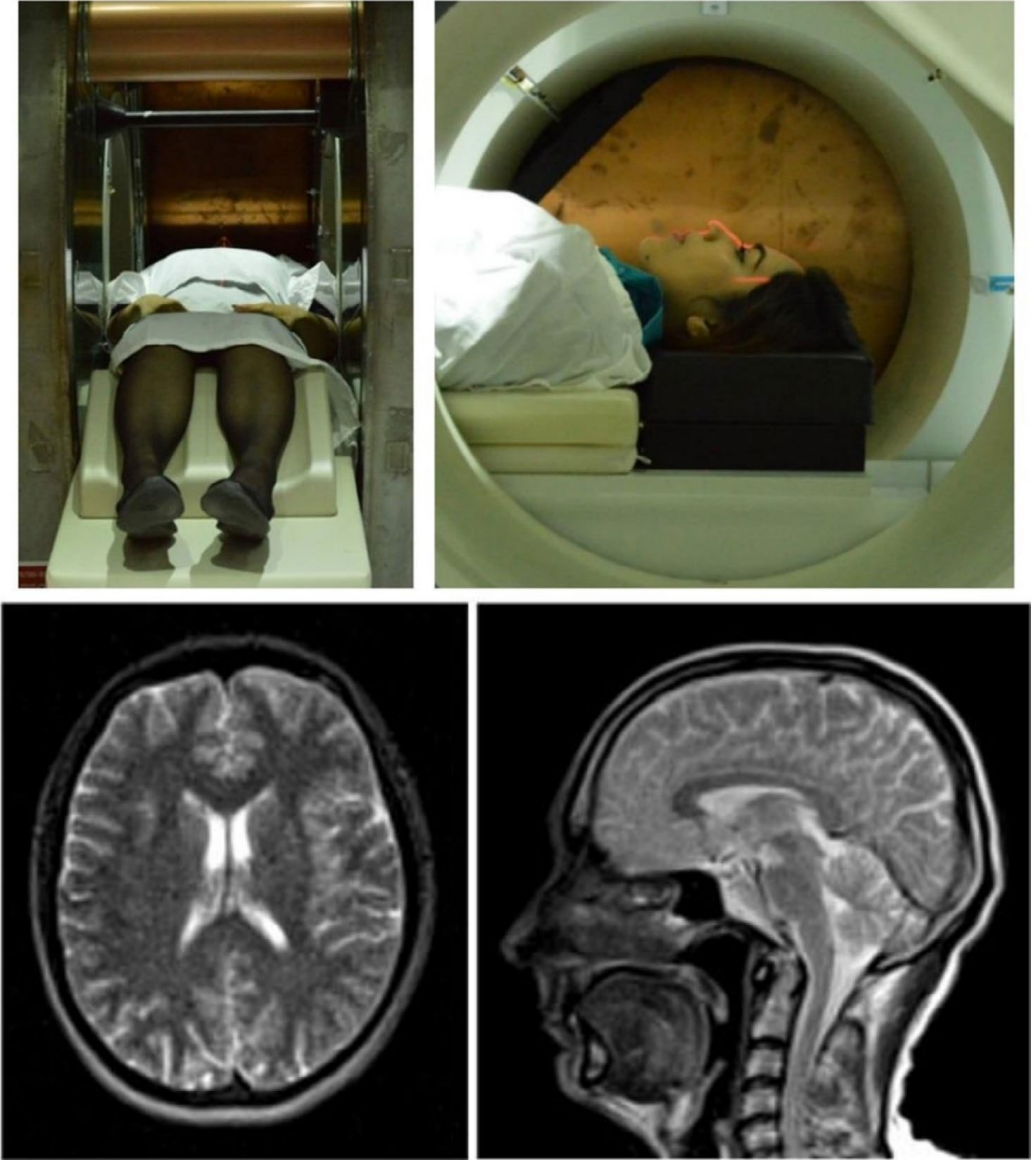
First in vivo images, March 2017 [20] with permission. Volunteer lying in the gap between the magnets (top) and the images of the brain obtained in the first in vivo experiment (bottom).

The Agilent 1 T magnet was delivered in April 2016. It just fitted through the bunker door with centimetres to spare (Figure 4). By March 2017, we had obtained the first in vivo images (Figure 5).

### 1.5 Research Program

The project comprised a partnership between six Australian universities: UNSW Sydney (clinical and physics), the University of Sydney (physics), the University of Queensland (MRI engineering), the University of Newcastle (dosimetry), the University of Wollongong (dosimetry), and Western Sydney University (MRI spectroscopy). The Liverpool Cancer Therapy Centre developed a program in MRI simulation to complement the MRI linac program.

Led by the Image X team at the University of Sydney, the program developed 4D modelling tools [21], advanced multileaf collimator tracking [22], and chaired an international guideline document [23] and a multi‐institutional review of MRI‐guided radiation therapy [24]. With the revolution in artificial intelligence (AI), the University of Sydney team integrated a range of real‐time AI technologies directly onto the MRI linac to improve beam adaptation and target tracking [25, 26, 27, 28]. These real‐time AI efforts emphasise fast image enhancement, motion estimation, and prediction to make soft‐tissue changes visible as they happen and support on‐the‐fly beam adjustments that ultimately aim to tighten margins and improve targeting without adding complexity to treatments.

**FIGURE 6:**
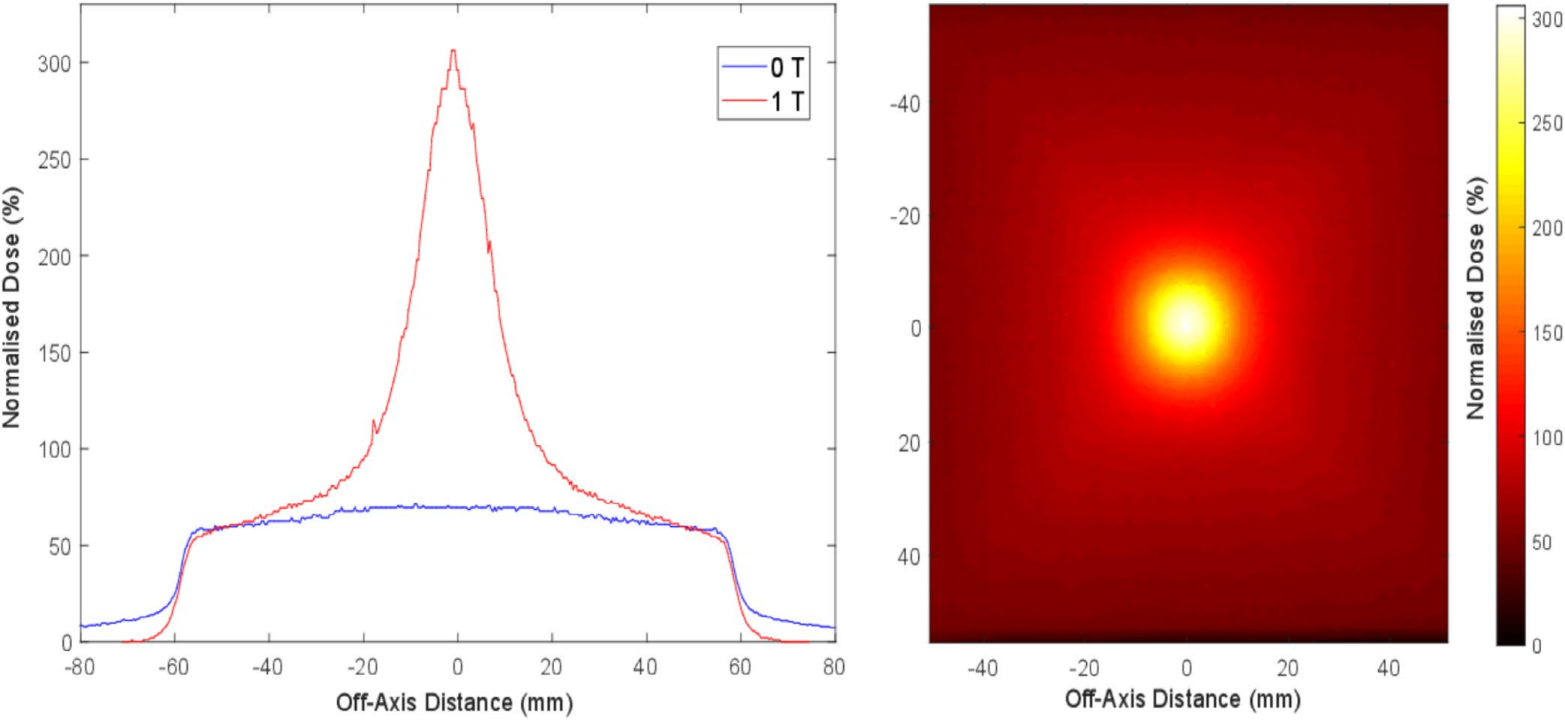
Electron hot spot. Dose vs. magnetic field strength in Monte Carlo modelling [30] (with permission).

The Centre for Medical Radiation Physics (CMRP) at the University of Wollongong focused on the development of robust radiation dosimetry in magnetic fields. The dosimeters employed included the MOSkin developed at CMRP in combination with commercially available radiochromic film [29]. The dosimeters characterised the skin dose due to electron streaming effects. A phenomenon first modelled by CRMP with Monte Carlo methods [16], this led to an electron focusing hot spot on the inline Australian MRI linac (Figure 6) [30]. The hot spot was ameliorated by electron steering magnets [31]. The dosimetry methods developed at the Australian MRI linac were essential to enable CMRP to characterise an electron streaming effect present on commercial transverse field MRI linacs known as the electron return effect. Electron streaming may occur in positions outside the treatment field and hence may require bolus or shielding. The CMRP team characterised this effect on several commercial MRI linacs [32]. This pioneering dosimetry formed important benchmark data for comparison with current planning systems' prediction of dose to patients and contributed to the development of Australasian MRI linac dosimetry guidelines [33].

The team at Western Sydney University (WSU) contributed their range of high‐field Nuclear Magnetic Resonance (NMR) spectrometers, ranging from 11.7 T to 14.1 T, to help define new potential targets for clinical strength MRI to aid the development of physiological targeting of individual cancers [34]. The high static magnetic field of the NMR afforded much greater sensitivity. Techniques and theory developed on high‐field machines can ultimately be translated to clinical field strengths. MRI provides diagnostic and targeting capabilities by detecting magnetic resonance‐detectable biomarkers that allow delineation between normal and neoplastic tissue. Such biomarkers can include differences in composition, structure or molecular dynamics, including reorientational and translational motion of molecules in the tissue types. For example, differences in reorientational motion are reflected in altered nuclear spin relaxation in *T*_1_‐weighted imaging and differences in translational motion are detected in diffusion‐weighted imaging. Most of these biomarkers have endogenous origins, but it is also possible to inject specialised compounds (’contrast agents’) that assist in delineating tissue types.

**FIGURE 7:**
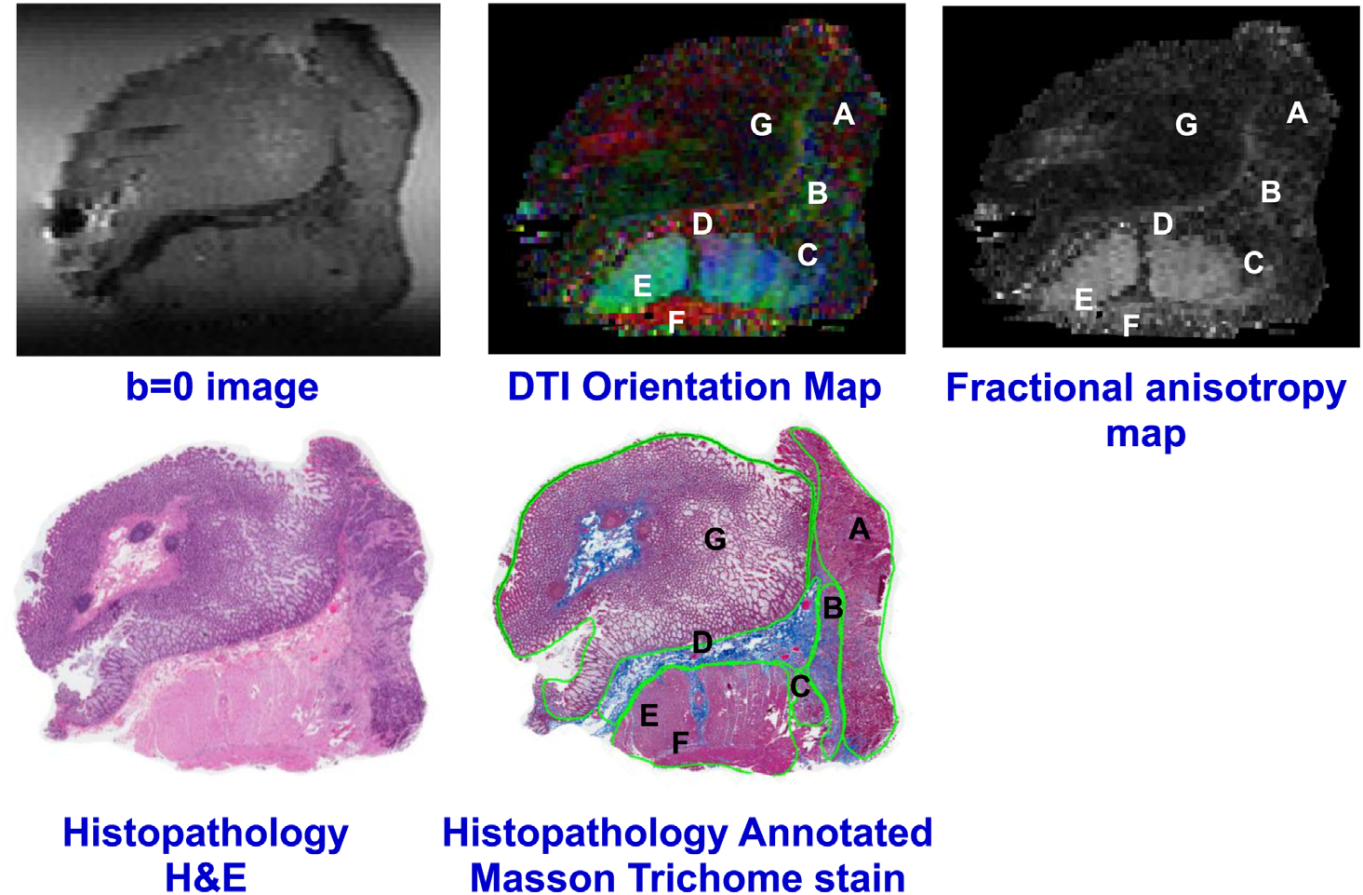
Correlation of high field MRI images with histopathology in human rectal cancer specimens. A = cancer, B+ desmoplasia, C = cancer invasion into desmoplasia. D = fibrous tissue, E = inner muscularis propria, F = outer muscularis propria, G = mucosa [35] (with permission).

The development of such biomarkers and their optimisation depend on hardware and theoretical developments. The theoretical developments can include improved pulse sequences (the instruction sets that control MRI machines in acquiring MRI images) and image reconstruction and analysis to tease out more information from the MRI data, including aspects such as cancer heterogeneity. Of course, the MRI data must be correlated and compared with ground truth histopathology during the development phase. The high‐field WSU facility was used to explore biomarkers of tumour heterogeneity in rectal cancer specimens by correlating MRI findings with histopathology [35] (Figure 7).

The University of Newcastle's contribution to the MRI linac program focused on methods to verify and ensure accurate treatments, predominantly developed by funded PhD student projects. The predominant focus was to develop quality assurance methods for MRI linac treatments to ensure safe and accurate treatment deliveries.

In vivo dosimetry methods using electronic portal imaging device (EPID) transit images were applied to the MRI linac. These were initially tested and validated on a conventional linac before experiments were performed with a research imager on the MRI linac. The performance of the EPID on the MRI linac in the presence of the magnetic field was examined and found to perform effectively. Experiments showed that EPID‐based estimation of doses in the phantom and hence the patient was feasible [36].

Novel methods to ensure quality and accuracy of synthetic CT generation for MRI‐only planning were developed and tested: EPID‐based transit dosimetry, a water‐equivalent depth method and a bulk‐density calculation method. The first method to use transit EPID images to validate synthetic CT generation methods was developed and tested with MRI‐only patient treatment data [37]. The method to optimise tissue, bone and fat densities for quality assurance dose calculations subsequently has been utilised for two multi‐centre trials of MRI‐only prostate planning in Australia for over 200 patients [38].

To take full advantage of MRI‐guided radiotherapy, we needed to integrate MRI into the full simulation and treatment workflow of a patient. A Siemens 3 T diagnostic MRI (MAGNETOM Skyra, Siemens Healthineers) was installed in the Liverpool Cancer Therapy Centre in 2013 to provide MRI simulation [39]. The centre has demonstrated leadership in MRI‐guided radiotherapy, with over 8500 patients scanned and a sustained utilisation rate of approximately 63% [40]. MR simulation has become integral to the planning of radical RT, complementing CT and enabling the use of MRI‐only planning for prostate patients within the NINJA trial. The implementation of advanced techniques such as 4D MRI and GRASP VIBE free‐breathing protocols has addressed the challenges of motion management and improved patient comfort, particularly in liver and abdominal SBRT [41]. The adoption of AI‐based technologies like Deep Resolve has further enhanced image quality and workflow efficiency.

### 1.6 Animal and Human Studies

A novel nano‐particle radiosensitiser with strong MR contrast properties was examined using a rat glioma model [42]. Animals were treated on the Australian MRI linac. Survival was not increased compared with control animals, but the agent was well tolerated, and tumour enhancement was clearly seen.

The Australian MRI linac clinical translation program consisted of three studies: A healthy volunteer MRI study, the Australian MRI linac patient imaging trial (AMPI), and the Australian MRI linac patient treatment trial (MANTRA).

A unique capability of the Australian MRI linac was the ability to do MRI‐guided multi‐leaf collimator (MLC) tracking of the tumour target. Patients were scanned on the Australian MRI linac as part of the AMPI trial. The patient data were used to simulate MRI‐guided multi‐target MLC tracking for 2 thorax cancer patients. One patient had recurrent thymoma with 2 left pleural‐based soft tissue metastases. A second patient had two primary non‐small cell lung cancers in the left upper lobe and right upper lobe, with mediastinal lymph node metastases. Coronal and sagittal cine‐MR images were able to track multiple targets clearly for both patients. The MR images were used to simulate multi‐target tracking, with an MLC field divided into two segments and the segmented MLC field moving independently in response to tumour motion on the cine‐MR images. These were recombined into a single deliverable MLC field. Multi‐target MLC tracking was shown to successfully target 2 targets independently during the simulated treatments for both patients, with coverage of 2 planning target volumes maintained.

A second unique capability of the Australian MRI linac is the ability to perform upright radiotherapy, which may be better tolerated in some patients with respiratory restriction. Upright and supine lung images were acquired on healthy volunteers. The results showed that volunteers in the upright position had a larger (19% increase) lung volume compared to the supine position.

We recruited two patients for the MANTRA trial for patients with brain metastases as the initial treatment site. The first patient had non‐small cell lung cancer (NSCLC) metastasis in the cerebellum only and was planned for partial brain (posterior fossa) radiotherapy on the Australian MRI linac. The main limitations of the Australian MRI linac were the restrictions on patient weight on the rotating couch and the bore size limitation, which excluded larger patients, and the inability of some cancer patients to tolerate the prone position. Due to patient ineligibility and technical limitations, no patients were treated on the Australian MRI Linac.

### 1.7 Achievements

The Australian MRI linac had many academic and professional achievements, including papers, PhD completions, grants, and patents. The program received over $27 million in competitive grant funding. Among the most sizable grants were the original HHF infrastructure grant, two consecutive NHMRC program grants spanning ten years, the Australian Cancer Research Foundation infrastructure grant, and grants from the Cancer Institute NSW and the NSW Cancer Council. Three early‐career fellowships were awarded to members of the research team.

The program produced over 120 per‐reviewed publications, and 25 PhDs were completed. Liverpool's multidisciplinary teams have contributed significantly to MRI in RT research and development, including the world's first MR in RT textbook [43]. Liverpool was the first Australian department to install a dedicated MRI simulator, as it was the first to install a dedicated CT simulator in 1995 [44]. The program has trained many radiation oncology physicists and therapists in MRI who have gone on to work in Australian radiotherapy departments as commercial MRI linacs became available. Dosimetry systems developed on the Australian MR linac led to successful use in quantifying the Electron Return effect and electron streaming on commercial MRI linacs [45].

The MRI linac program sponsored the Australian MR in RT annual conference, which became a focus for the development of MRI in radiotherapy more broadly and facilitated clinical research in partnership with the Trans Tasman Radiation Oncology Group.

### 1.8 What Went Wrong

Of course, the MRI linac program was not all plain sailing. The temporary Siemens MRI suffered a containment breach and vented its helium. A fire broke out in the Varian Linatron Modulator, which triggered the sprinkler system—both fire and flood!

The installation and decommissioning of the magnet resulted in significant beam configuration changes in the adjacent treating radiotherapy bunkers, requiring intervention.

Finally, the program failed to develop into a commercial product. The number of potential commercial partners was limited to two accelerator manufacturers, Varian and Elekta. Both had programs with other MRI linac prototypes. The design presented a number of practical limitations, including patient tolerance of rotation [15], the couch weight tolerance, and the size of the patient that could be accommodated in the split.

## 2 Discussion

The Australian MRI linac program grew from a desire to create a world‐leading medical physics project that would attract research physicists and provide a focus for training and development of academic medical physics in a clinical setting. Scientifically, it contributed to the knowledge about MRI guidance, including novel designs, radiation interactions with magnet fields, patient tracking and radiation dosimetry in high magnetic fields. The MRI linac program introduced MRI guidance to the Australian clinical practice by training clinical staff on its application. It was the first in Australia to develop in‐house MRI simulation and completely integrate MRI into clinical radiotherapy practice.

Academically, the program was successful in generating highly cited publications, research fellowships, PhDs in physics and medicine, and obtaining competitive grant support. The failure of the program to result in a commercial product does not negate its major contribution to the field of MRI guidance or to the development of academic medical physics in Australia. The program initiated the annual Australian MR in RT conference. A strong program in medical physics research has been sustained at Liverpool Hospital. The investigators chaired the development of international guidelines on MRI‐guided radiotherapy.

The MRI linac program is a good example of a big idea in research. Big ideas aim to meet an unmet need for a tractable health problem of national or international significance. They focus on the entity, not individual projects. Large calls for funding frequently appear out of the blue, and successful applicants are those who have developed research programs over time rather than cobbled together at the call for funding. The Australian MRI linac program seized the opportunity presented by the HHF in 2008 because it was a well‐developed concept to create a focus of medical physics research at the Liverpool CTC. It created a major centre for the next step in radiotherapy image guidance and examined a novel implementation. The program combined world‐class physics, engineering and physical science facilities at six Australian Universities with an expanding clinical cancer service that was willing to support its implementation. The radiation oncology community in Australia and New Zealand has an extensive history of collaborative research, particularly with clinical trials, that could be used as the basis of new technological programs such as MRI‐guided proton therapy [46].

The central promise of MRI linac technology has long been that better imaging enables tighter margins and more conformal dose delivery. However, advances in image‐guided radiotherapy have demonstrated a law of diminishing returns, with plan quality plateauing even on the most advanced systems [47]. The Australian MRI linac Program identified new avenues through which MRI guidance can provide meaningful clinical benefit.

Firstly, real‐time volumetric MRI is essential for tracking tumour motion during beam delivery. The Australian MRI linac Program researchers demonstrated the integration of deep learning–powered super‐resolution imaging on treatment machines to accelerate MRI‐based motion tracking—an essential capability for future adaptive workflows [26, 27].

Secondly, the economic barriers to MRI linac deployment are likely to fall as low‐field technologies mature. Both ViewRay and the Australian MRI linac operate below diagnostic field strengths, demonstrating that ≥ 1.5 T is not essential for guidance. Portable ≤ 0.5 T MRI systems promise further reductions in cost and footprint [48].

Thirdly, quantitative MRI will support biologically guided dose modulation. The Australian MRI linac led to a prospective study using oxygen‐enhanced MRI to identify hypoxic subregions in glioblastoma [49], while related work in multiparametric planning suggests dose painting is technically feasible and may improve outcomes [50].

In the longer term, physics continues to push toward real‐time dose verification. The Australian MRI linac Program demonstrated 3D gel‐based dosimetry on the MRI linac in phantoms [51]. Although significant innovation is required, translating this to clinical use would close the loop between prescription, delivery, and verification. Together, these avenues point to a future for MRI in radiotherapy that goes beyond dose conformity and leads to MRI linacs becoming an integral component of cancer treatment.

## 3 Conclusion

The learnings from the Australian MRI linac program are that Australia has world‐class radiotherapy research in physics and engineering, that major projects need a lot of time and a lot of collaboration, and that large novel radiotherapy projects in Australia can attract significant funding and produce significant results.

## Author Contributions

**Michael Bernard Barton:** conceptualization, methodology, project administration, writing – original draft, writing – review and editing, funding acquisition. **Lois Holloway:** conceptualization, investigation, methodology, formal analysis, supervision, resources, writing – original draft, writing – review and editing, project administration. **William S. Price:** conceptualization, investigation, methodology, validation, formal analysis, supervision, resources, writing – review and editing, writing – original draft, funding acquisition, software. **David Waddington:** conceptualization, investigation, methodology, writing – review and editing, writing – original draft, formal analysis, project administration. **Geoffrey Paul Delaney:** conceptualization, investigation, funding acquisition, writing – original draft, writing – review and editing, project administration, resources, supervision. **Robba Rai:** conceptualization, investigation, formal analysis, writing – original draft, writing – review and editing, methodology, validation, funding acquisition. **Peter Greer:** investigation, methodology, writing – review and editing, writing – original draft, conceptualization, formal analysis, supervision, resources. **Paul Keall:** conceptualization, investigation, funding acquisition, writing – original draft, writing – review and editing, methodology, formal analysis, software, supervision, resources, project administration. **Peter Metcalfe:** conceptualization, investigation, methodology, formal analysis, supervision, writing – review and editing, writing – original draft, project administration. **Trang Pham:** conceptualization, investigation, methodology, formal analysis, writing – review and editing, writing – original draft. **Stuart Crozier:** conceptualization, investigation, funding acquisition, writing – original draft, methodology, validation, writing – review and editing, project administration, resources, supervision.

## Funding

This work was supported by the National Health and Medical Research Council (APP1132471 and APP1036078), the Australian Research Council, the Cancer Institute NSW, the Australian Cancer Research Foundation and the Department of Health and Ageing, Australian Government.

## Conflicts of Interest

The authors declare no conflicts of interest.

## Data Availability

Data sharing not applicable to this article as no datasets were generated or analysed during the current study.
